# Strategies for formula constant optimisation for intraocular lens power calculation

**DOI:** 10.1371/journal.pone.0267352

**Published:** 2022-05-05

**Authors:** Achim Langenbucher, Nóra Szentmáry, Alan Cayless, Jascha Wendelstein, Peter Hoffmann

**Affiliations:** 1 Department of Experimental Ophthalmology, Saarland University, Homburg/Saar, Germany; 2 Dr. Rolf M. Schwiete Center for Limbal Stem Cell and Aniridia Research, Saarland University, Homburg/Saar, Germany; 3 Department of Ophthalmology, Semmelweis-University, Budapest, Hungary; 4 School of Physical Sciences, The Open University, Milton Keynes, United Kingdom; 5 Department of Ophthalmology, Johannes Kepler University Linz, Linz, Austria; 6 Augen- und Laserklinik Castrop-Rauxel, Castrop-Rauxel, Germany; Edith Wolfson Medical Center, ISRAEL

## Abstract

**Background:**

To investigate modern nonlinear iterative strategies for formula constant optimisation and show the application and results from a large dataset using a set of disclosed theoretical-optical lens power calculation concepts.

**Methods:**

Nonlinear iterative optimisation algorithms were implemented for optimising the root mean squared (SoSPE), the mean absolute (SoAPE), the mean (MPE), the standard deviation (SDPE), the median (MEDPE), as well as the 90% confidence interval (CLPE) of the prediction error (PE), defined as the difference between postoperative achieved and formula predicted spherical equivalent power of refraction. Optimisation was performed using the Levenberg-Marquardt algorithm (SoSPE and SoAPE) or the interior point method (MPE, SDPE, MEDPE, CLPE) for the SRKT, Hoffer Q, Holladay 1, Haigis, and Castrop formulae. The results were based on a dataset of measurements made on 888 eyes after implantation of an aspherical hydrophobic monofocal intraocular lens (Vivinex, Hoya).

**Results:**

For all formulae and all optimisation metrics, the iterative algorithms showed a fast and stable convergence after a couple of iterations. The results prove that with optimisation for SoSPE, SoAPE, MPE, SDPE, MEDPE, and CLPE the root mean squared PE, mean absolute PE, mean PE, standard deviation of PE, median PE, and confidence interval of PE could be minimised in all situations. The results in terms of cumulative distribution function are quite coherent with optimisation for SoSPE, SoAPE, MPE and MEDPE, whereas with optimisation for SDPE and CLPE the standard deviation and confidence interval of the PE distribution could only be minimised at the cost of a systematic offset in mean and median PE.

**Conclusion:**

Nonlinear iterative techniques are capable of minimising any statistical metrics (e.g. root mean squared or mean absolute error) of any target parameter (e.g. PE). These optimisation strategies are an important step towards optimising for the target parameters which are used for evaluating the performance of lens power calculation formulae.

## Introduction

The refractive power of intraocular lenses can be calculated using either empirical formulae, or so called theoretical-optical formulae based on deep learning algorithms, or with combinations [1, 2] of these. With the SRK or SRK2 formula [1, 3], the power of the intraocular lens (IOL) for emmetropisation is derived from the corneal power (calculated from the corneal radius using a keratometer index (n_K_) of 1.3375), axial length (AL) and a formula constant (A), which adapts the formula to a specific lens design. In addition to this simplistic regression formula, several theoretical-optical formulae have been published based on a pseudophakic eye model, in which the IOL power for emmetropisation is based on: the AL, the corneal power calculated from corneal curvature with a keratometer index, other optional biometric measures such as anterior chamber depth (ACD), central thickness of the cornea (CCT) or the crystalline lens (LT), the horizontal corneal diameter (W2W), the age of the patient, and one or more formula constants which again adapt the generally defined formula to the characteristics of a specific lens design [1, 4]. The classical calculation concepts use formula constants A (SRKT formula [3, 5]), personalised anterior chamber depth pACD (Hoffer Q formula [6–8]), surgeon factor SF (Holladay 1 formula [9]), or constant triplets a0/a1/a2 (Haigis formula [1]). All of these theoretical optical formulae based on the Gernet or Fyodorov (published independently in 1970 [10] and 1975 [11]) are restricted to linear Gaussian optics (paraxial optics). The prediction of the axial position of the thin IOL implant (effective lens position, ELP) is mostly performed empirically using the formula constant [12–14]. In addition to these classical calculation concepts, over the last 2 or decades many IOL power calculation strategies such as the Holladay 2, the Barrett, Kane, T2, or DGS formula have been presented, and their relative merits discussed in many scientific reports [15–17]. Only a few of these modern IOL power calculation formulae (for example, the Castrop formula) have been disclosed by the formula authors [16, 18]. In contrast to all these formulae, IOL power calculations using deep learning (e.g. the Hill RBF calculator) are based on a big data setup instead of an eye model, and they do not require formula constants to consider the specific characteristics of a lens model.

It is well known that formula constants are specific not only to IOL models, but also to the clinical population, the surgical technique, and to the measurement techniques (e.g. refractometry) or the equipment (e.g. the biometer). Therefore formula constants should be customised to the surgeon or the surgical centre [19, 20]. For optimisation of formula constants, in addition to the preoperative biometric measures which are considered in the lens power calculation formula, the power of the IOL inserted in the patient eye and the achieved refraction (spherical equivalent, SEQ) after cataract surgery are required. Special attention should be given to the postoperative refraction, which should be documented in a time interval at least 4 weeks after cataract surgery using manual refractometry using trial glasses in a trial frame. Eyes with a postoperative Snellen decimal visual acuity lower than 0.8 should not be considered as the refraction might be unreliable.

However, there is in general no consensus on the optimisation technique for formula constants [18–20]. If the IOL power calculation formula is fully disclosed and uses only one formula constant, the formula can be reorganised to solve for the formula constant. For each individual eye a formula constant is extracted, combining the preoperative biometric measures, the power of the implanted IOL, and the postoperative refraction. Ultimately, any statistical measure such as the mean or median could be used to identify the proper formula constant for this study population from the formula constant distribution. However, when using this ‘optimised’ formula constant in a back-calculation procedure, due to nonlinearity of the formula, we have to be aware that such a procedure does not necessarily result in a zero mean or median prediction error for the spherical equivalent, the least mean absolute prediction error (MAE), the least root mean squared prediction error (RMS), or the least confidence intervals (CL) in the distribution of the prediction error [19]. With more than 1 formula constant in the calculation concept, a direct back calculation of the formula constants for an individual patient eye is not possible. In the simplest case, if all the formula constants together define the ELP in a multilinear superposition, the formula can be solved for the ELP and the formula constants retrieved with a multivariable linear regression analysis from the ELP (e.g. a0/a1/a2 with the Haigis formula). In IOL calculation concepts with more than one formula constant which do not define the ELP with a multilinear superposition (such as the Castrop formula), optimisation procedures fail, and nonlinear optimisation strategies have to be applied [18].

The **purpose of the present study** is to present a methodology for nonlinear iterative optimising formula constants:

- which could be used for any fully disclosed IOL power calculation concept, and
- which is in general capable of minimising any statistical measure (e.g. mean, median, standard deviation, confidence interval, or combinations) of any target parameter (e.g. SEQ prediction error).

This optimisation strategy is applied to a large dataset of measurements made after cataract surgery from one clinical centre using one IOL type, in order to explain the meaning of the formula constant optimisation results more in detail.

## Materials and methods

### Dataset for formula constant optimisation

In this retrospective study we analysed a dataset containing measurements from 888 eyes from a cataract population from Augen- und Laserklinik Castrop-Rauxel, Castrop-Rauxel, Germany which was transferred to us (490 right eyes and 398 left eyes; 495 female and 392 male). The mean age was 71.2±9.1 years (median: 71 years, range: 47 to 91 years). The study was registered with the local ethics committee (Ärztekammer des Saarlandes, registration number 157/21), and a patient informed consent was not required for this study. The data were transferred to us in an anonymised fashion, which precludes back-tracing of the patient. The anonymised data contained preoperative biometric data derived with the IOLMaster 700 (Carl-Zeiss-Meditec, Jena, Germany) including axial length AL, anterior chamber depth ACD measured from the corneal front apex to the anterior apex of the crystalline lens, lens thickness LT, and the corneal front surface radius measured in the flat (R1) and in the steep meridian (R2). In all cases a Vivinex 1 piece hydrophobic aspherical (aberration correcting) monofocal intraocular lens (Hoya Surgical Optics, Singapore) was inserted. In addition to the refractive power of the inserted lens (PIOL), the postoperative refraction (sphere and cylinder) 5 to 12 weeks after cataract surgery was measured by an experienced optometrist and recorded in the dataset. The dataset included only data with a postoperative Snellen decimal visual acuity of 0.8 (20/25 Snellen lines) or higher to ensure that the postoperative refraction was reliable. The descriptive data on pre-cataract biometry, PIOL and postoperative refraction are summarised in **Table 1**.

**Table 1:** Descriptive statistics of the dataset with mean, standard deviation (SD), median, and the lower (quantile 5%) and upper (quantile 95%) boundary of the 90% confidence interval.

N = 888	AL in mm	ACD in mm	LT in mm	R1 in mm	R2 in mm	Rmean	PIOL in dpt	SEQ in dpt
Mean	24.10	3.19	4.62	7.85	7.67	7.77	20.62	-0.56
SD	1.41	0.41	0.46	0.28	0.27	0.27	3.73	0.92
Median	23.90	3.18	4.59	7.85	7.67	7.77	21.0	-0.25
Quantile 5%	22.10	2.51	3.86	7.40	7.22	7.31	13.5	-2.38
Quantile 95%	26.78	3.83	5.36	8.33	8.15	8.23	26.0	0.38

AL refers to the axial length, ACD to the external phakic anterior chamber depth measured from the corneal front apex to the front apex of the crystalline lens, LT to the central thickness of the crystalline lens, R1 and R2 to the corneal radius of curvature for the flat and steep meridian, Rmean to the average of R1 and R2, PIOL to the refractive power of the intraocular lens implant, and SEQ to the spherical equivalent power achieved 4 to 12 weeks after cataract surgery.

The anonymised Excel data (.xlsx-format) was imported into MATLAB to Matlab (Matlab 2019b, MathWorks, Natick, USA) for further processing.

### Preprocessing of the data

Custom software was written in Matlab. The patient age was derived from the date of cataract surgery and date of birth. The mean corneal radius of curvature Rmean was calculated as Rmean = ½(R1+R2), and the mean corneal power Kmean was derived from R1 and R2 as Kmean = ½((n_K_-1)/R1+(n_K_-1)/R2) with a keratometer index n_K_ as indicated in the formula definition. The following lens power calculation formulae were considered in this constant optimisation process:

- SRKT formula published by Sanders, Retzlaff and Kraff [3, 5],
- Hoffer Q formula published by Hoffer [6–8],
- Holladay 1 formula published by Holladay and Prager [9],
- Haigis formula [1], as well as the
- Castrop formula published by Wendelstein et al. and Langenbucher et al. [16, 18].

The SRKT, Hoffer Q, and Holladay 1 formulae consider the AL and corneal curvature/power data together with one formula constant (A, pACD, and SF, respectively), the Haigis formula considers the AL, ACD, and corneal curvature together with a formula constant triplet a0/a1/a2s, and the Castrop formula considers AL, CCT, ACD, LT and corneal curvature of the front and back surface together with a formula constant triplet C/H/R. For the Haigis formula we used 2 versions: the simplified option (Haigis1) with preset values a1 = 0.4/a2 = 0.1 and customisation of a0, and additionally the option with a customised formula constant triplet a0/a1/a2 (Haigis3) [19]. For simplicity when using the Castrop formula, instead of using the CCT and the curvature data of the corneal back surface, we replaced CCT by 0.55 mm and the corneal back surface curvature was estimated from the corneal front surface curvature with a fixed anterior to posterior curvature ratio of 0.834 as published by Langenbucher et al. [18], and a sum of segments correction according to Cooke [21, 22] was used for the axial length. All formulae included in this analysis were reorganised and solved for the SEQ as a function of preoperative biometrical data and PIOL. The difference between the achieved SEQ (from the postoperative follow-up examination) and the SEQ predicted by the formula was considered as the formula prediction error PE.

### Formula constant optimisation

In this context, formula constants were optimised for statistical metrics of the SEQ prediction error PE. For the statistical metrics we used: the sum of squared PE (SoSPE) which minimises the ‘energy’ of the prediction error, the sum of the absolute PE (SoAPE, as typically used in scientific reports on the performance of lens power prediction formulae), zeroing of the mean the PE (MPE), the standard deviation of the PE (SDPE) and a linear combination of both (SDMPE), and zeroing of the median PE (MEDPE), the 90% confidence interval (CLPE) and combination of both (CLMEDPE). For all formulae under test (SRKT, Hoffer Q, Holladay 1, Haigis1, Haigis3, and Castrop) formula constant optimisation programming code was written in Matlab for the above mentioned metrics.

For optimisation of the PE for minimising the SoSPE and SoAPE we used the Levenberg-Marquardt algorithm, which is also quoted in the literature as a damped least-squares method and in general solves non-linear least squares problems. This optimisation technique was first described by Levenberg in 1944 [23] and 2 decades later rediscovered by Marquardt [24]. This algorithm is typically used in least squares curve fitting [25] and in most cases converges faster than simple back-propagation methods. It combines the classical Gauss-Newton algorithm with the gradient descent algorithms and is–in most applications–more robust in terms of finding the global minimum, with the potential drawback that the convergence may be somewhat slower compared to the Gauss-Newton algorithm.

Formula constant optimisation for MPE, SDPE, SDMPE, MEDPE, CLPE, and CLMEDPE was implemented using the interior point methods, which refer to a family of optimisation techniques for solving linear and nonlinear convex optimisation problems. This algorithm is also quoted as the barrier method. First published by Dikin in 1967 [26] and 2 decades later re-discovered by Karmarker [27], this algorithm is very efficient in terms of minimising the number of iterations and the number of function evaluations, but seems to be less robust in searching a global minimum [28]. It can be directly implemented with linear programming techniques, and in many situations shows a better performance compared to the simplex algorithm.

Both optimisation techniques–the Levenberg-Marquardt algorithm [23, 24] and the interior point method [26, 27]–were used with box constraint boundaries. The formula constants used for initialisation were: 118.9 (boundaries 116.0 to 121.0) for the SRKT formula, pACD = 5.4 (boundaries from 4.5 to 6.3) for the Hoffer Q, SF = 1.5 (boundaries from 0.5 to 3.0) for the Holladay, a0 = 1.8 (boundaries from -1.0 to 2.5) for the Haigis1, a0/a1/a2 = 1.8/0.4/0.1 (boundaries from -1.0 to 2.5/0.0 to 0.8/0.0 to 0.3 for the Haigis3, and C/H/R = 0.4/0.0/0.0 (boundaries from 0.25 to 0.45/-0.35 to 0.35/-0.35 to 0.35 for the Castrop formula, respectively. After optimising the formula constants for SoSPE, these constants were used as presets for all other optimisations, and the respective boundaries were set symmetrically to ±0.5, ±0.5, ±0.5, ±0.5, ±0.5/±0.1/±0.1, and ±0.1/±0.35/±0.35, respectively. The box constraints were not reached in any iteration step of any of the optimisations.

The SEQ prediction was back-calculated using the optimised constants for each formula and each optimisation metric, and the prediction error PE was derived and analysed. The performance of the formula constant optimisation process was documented with the same metrics which were used earlier for the constant optimisation (sum of squared PR, sum of absolute PE, mean PE, SD of PE, median PE, and 90% CL of PE).

## Results

In general, the convergence of the iterative optimisation process for all formulae was faster when optimising for the sum of squared PE (up to 26 iterations and 152 function evaluations), the mean PE (up to 30 iterations and 148 function evaluations) or the median PE (up to 32 iterations and 177 function evaluations), as compared to the optimisation for the sum of absolute PE (up to 48 iterations and 255 function evaluations) standard deviation (up to 50 iterations and 311 function evaluations), 90% confidence interval (up to 48 iterations and 198 function evaluations) or combinations of standard deviation and mean or 90% confidence interval and median PE (up to 77 iterations and 510 function evaluations). Convergence was achieved in all optimisations for all formulae under test.

The resulting optimised formula constants in terms of minimising the sum of squared prediction error (SoSPE), sum of absolute prediction error (SoAPE, mean prediction error (MPE), standard deviation of prediction error (SDPE), combinations of standard deviation and mean prediction error (SDMPE), median prediction error (MEDPE), 90% confidence interval of prediction error (CLPE), and combinations of 90% confidence interval of prediction error and median prediction error (CLMEDPE) are listed in **Table 2**.

**Table 2:** Optimised formula constants for the SRKT, the Hoffer Q, the Holladay 1, Haigis (with optimised a0 and preset values a1 = 0.4 / a2 = 0.1, Haigis1; and with optimised a0 / a1 / a2 constant triplet, Haigis3), and Castrop formula.

		SoSPE	SoAPE	MPE	SDPE	SDMPE	MEDPE	CLPE	CLMEDPE
SRKT	A	119.2748	119.2877	119.2698	119.3783	119.2698	119.2854	119.2810	119.2811
Hoffer Q	pACD	5.7356	5.7336	5.7638	5.4638	5.7638	5.7549	5.6517	5.6564
Holladay 1	SF	1.9618	1.9565	1.9762	1.6762	1.9762	1.9661	1.8683	1.9230
Haigis1	a0	1.5633	1.5540	1.5884	1.2884	1.5884	1.5934	1.5530	1.5702
Haigis3	a0	-0.6853	-0.8422	-0.6846	-0.3346	-0.6846	-0.6920	-0.6856	-0.6410
	a1	0.3417	0.3524	0.3420	0.3308	0.3420	0.3459	0.3526	0.3340
	a2	0.2029	0.2077	0.2030	0.1795	0.2030	0.2025	0.2024	0.2021
Castrop	C	0.2814	0.2517	0.2814	0.2746	0.2814	0.2780	0.2780	0.2780
	H	0.3500	0.5014	0.3500	0.3905	0.3500	0.3653	0.3477	0.3477
	R	0.0848	0.0554	0.0848	0.0848	0.0848	0.0765	0.1083	0.1019

Formula constant optimisation was performed to minimise the sum of squared prediction errors (SoSPE), the sum of absolute prediction errors (SoAPE), the mean prediction error (MPE), standard deviation of prediction error (SDPE), a combination of mean and standard deviation of prediction error (SDMPE), median prediction error (MEDPE), the 90% confidence interval of prediction error (CLPE), and a combination of median and 90% confidence interval of prediction error (CLMEDPE).

Except for the Castrop formula where all optimisation strategies yielded consistent results, an optimisation solely for the standard deviation or the 90% confidence interval of the prediction error may in some cases yield formula constants which deviate from those derived from an optimisation for sum of squared or sum of absolute prediction error, mean or median prediction error, or combinations of standard deviation and mean or confidence interval and median prediction error.

In the next step, the optimised formula constants for the 8 different metrics were used to derive the formula predicted refraction and to calculate the prediction error in terms of achieved spherical equivalent minus formula predicted refraction. In **Table 3**, the mean, standard deviation, median, lower and upper boundary of the 90% confidence interval together with the width of the 90% confidence interval, the mean absolute and the root mean squared prediction error are listed for all formulae under test.

**Table 3:** Prediction error (PE) as the difference between achieved and formula predicted spherical equivalent for 8 different statistical metrics of formula constant optimisation and various formulae under test.

N = 888; optimisation for →		SoSPE	SoAPE	MPE	SDPE	SDMPE	MEDPE	CLPE	CLMEDPE
SRKT	MEAN	-0.0041	-0.0147	**0.0000**	-0.0884	0.0000	-0.0128	-0.0092	-0.0092
	SD	0.4414	0.4413	0.4414	**0.4410**	0.4414	0.4413	0.4413	0.4413
	MEDIAN	0.0097	-0.0019	0.0143	-0.0775	0.0143	**0.0000**	*0*.*0034*	0.0033
	5% quantile	-0.7089	-0.7181	-0.7064	-0.7866	-0.7064	-0.7160	-0.7121	-0.7121
	95% quantile	0.7095	0.7002	0.7120	0.6298	0.7120	0.7020	0.7054	0.7054
	90% CL	1.4184	1.4184	1.4184	1.4164	1.4184	1.4181	**1.4175**	1.4175
	ABS	0.3407	**0.3405**	0.3408	0.3471	0.3408	0.3405	0.3406	0.3406
	RMS	**0.4412**	0.4413	0.4412	0.4495	0.4412	0.4412	0.4412	0.4412
Hoffer Q	MEAN	0.0370	0.0397	**0.0000**	0.3969	0.0000	0.0116	0.1474	0.1412
	SD	0.4275	0.4272	0.4307	**0.4027**	0.4307	0.4297	0.4185	0.4189
	MEDIAN	0.0291	0.0322	-0.0115	0.3941	-0.0115	**0.0000**	*0*.*1383*	0.1309
	5% quantile	-0.6550	-0.6512	-0.7016	-0.2541	-0.7016	-0.6876	-0.5326	-0.5404
	95% quantile	0.7702	0.7733	0.7330	1.0423	0.7330	0.7418	0.8385	0.8336
	90% CL	1.4252	1.4246	1.4346	1.2964	1.4346	1.4294	**1.3711**	1.3739
	ABS	0.3327	**0.3327**	0.3346	0.4657	0.3346	0.3336	0.3463	0.3449
	RMS	**0.4288**	0.4288	0.4305	0.5652	0.4305	0.4296	0.4435	0.4419
Holladay 1	MEAN	0.0188	0.0257	**0.0000**	0.3942	0.0000	0.0132	0.1410	0.0694
	SD	0.4256	0.4253	0.4265	**0.4169**	0.4265	0.4259	0.4210	0.4235
	MEDIAN	0.0057	0.0116	-0.0130	0.4034	-0.0130	**0.0000**	*0*.*1275*	0.0546
	5% quantile	-0.6576	-0.6497	-0.6784	-0.2710	-0.6784	-0.6640	-0.5251	-0.6011
	95% quantile	0.8011	0.8056	0.7905	1.0901	0.7905	0.7981	0.8876	0.8390
	90% CL	1.4588	1.4553	1.4689	1.3611	1.4689	1.4621	**1.4127**	1.4400
	ABS	0.3269	**0.3269**	0.3277	0.4689	0.3277	0.3271	0.3444	0.3296
	RMS	**0.4258**	0.4259	0.4262	0.5736	0.4262	0.4258	0.4438	0.4289
Haigis1	MEAN	0.0334	0.0458	**0.0000**	0.4033	0.0000	-0.0067	0.0471	0.0242
	SD	0.4027	0.4017	0.4055	**0.3789**	0.4055	0.4061	0.4015	0.4034
	MEDIAN	0.0341	0.0434	0.0071	0.3940	0.0071	**0.0000**	*0*.*0444*	0.0267
	5% quantile	-0.6472	-0.6331	-0.6853	-0.2350	-0.6853	-0.6929	-0.6316	-0.6578
	95% quantile	0.7140	0.7263	0.6843	1.0394	0.6843	0.6802	0.7276	0.7049
	90% CL	1.3612	1.3594	1.3696	1.2744	1.3696	1.3731	**1.3592**	1.3626
	ABS	0.3156	**0.3154**	0.3173	0.4580	0.3173	0.3177	0.3154	0.3159
	RMS	**0.4038**	0.4040	0.4053	0.5532	0.4053	0.4059	0.4041	0.4039
Haigis3	MEAN	0.0065	0.0172	**0.0000**	0.3305	0.0000	0.0108	-0.0247	0.0058
	SD	0.3711	0.3712	0.3712	**0.3677**	0.3712	0.3711	0.3717	0.3712
	MEDIAN	-0.0056	0.0024	-0.0126	0.3222	-0.0126	**0.0000**	*-0*.*0369*	-0.0062
	5% quantile	-0.5930	-0.5763	-0.5997	-0.2535	-0.5997	-0.5906	-0.6257	-0.5922
	95% quantile	0.6234	0.6439	0.6159	0.9440	0.6159	0.6254	0.5821	0.6151
	90% CL	1.2163	1.2202	1.2157	1.1976	1.2157	1.2160	**1.2077**	1.2073
	ABS	0.2830	**0.2826**	0.2833	0.3987	0.2833	0.2829	0.2852	0.2833
	RMS	**0.3710**	0.3714	0.3710	0.4943	0.3710	0.3710	0.3724	0.3710
Castrop	MEAN	0.0000	0.0114	**0.0000**	-0.0120	0.0000	0.0089	0.0008	0.0072
	SD	0.3437	0.3440	0.3437	**0.3437**	0.3437	0.3437	0.3438	0.3438
	MEDIAN	-0.0075	0.0000	-0.0075	-0.0205	-0.0075	**0.0000**	*-0*.*0064*	0.0000
	5% quantile	-0.5626	-0.5372	-0.5626	-0.5730	-0.5626	-0.5530	-0.5556	-0.5492
	95% quantile	0.5553	0.5692	0.5553	0.5461	0.5553	0.5643	0.5554	0.5618
	90% CL	1.1180	1.1064	1.1180	1.1191	1.1180	1.1173	**1.1111**	1.1111
	ABS	0.2662	**0.2658**	0.2662	0.2666	0.2662	0.2660	0.2661	0.2661
	RMS	**0.3435**	0.3440	0.3435	0.3437	0.3435	0.3436	0.3436	0.3436

MEAN/SD/MEDIAN/5%quantile/95%quantile/90%CL/ABS/RMS refer to mean/standard deviation/median/5% and 95% quantile/90% confidence interval/mean absolute/root mean squared prediction error (PE). Columns 2 to 9 refer to formula constant optimisation for least sum of squared PE (SoSPE), least sum of absolute PE (SoAPE), least mean PE (MPE), least standard deviation of PE (SDPE), combination of least mean and standard deviation of PE (SDMPE), least median PE (MEDPE), smallest 90% confidence interval of PE (CLPE), and a combination of least median and 90% confidence interval of PE. The numbers in bold indicate the PE values in each row that are expected to be the lowest based on the optimisation strategy. For example, the RMS PE / ABS PE would be expected to be lowest where optimisation was performed for the sum of squared prediction errors / sum of absolute PE. Optimisation for the standard deviation of prediction error SDPE resulted in a MEAN of up to 0.40 dpt (underlined numbers), and optimisation for the 90% confidence interval CLPE resulted in a MEDIAN of up to 0.14 dpt (italic numbers). All data are provided in dioptres.

The numbers marked in bold in each row indicate the prediction error corresponding to the optimisation metric for that row, and which is therefore expected to yield the lowest value. For example, in the row corresponding to optimisation for the mean or the median prediction error, the resulting mean or median prediction error would be expected to have the smallest value. In general, except when optimising for the standard deviation or the 90% confidence interval without considering the respective mean or median value, the optimisation metrics provide consistent results. When using the standard deviation or the 90% confidence intervals as the sole metrics for formula constant optimisation, the mean / median prediction error may in some cases have a systematic offset up to 0.4 dpt (underlined numbers) / 0.14 dpt (italic numbers). This systematic offset in mean / median prediction error could be mostly compensated for by using metrics for formula constant optimisation which consider combinations of the standard deviation and mean or 90% confidence interval and median.

**Fig 1:**
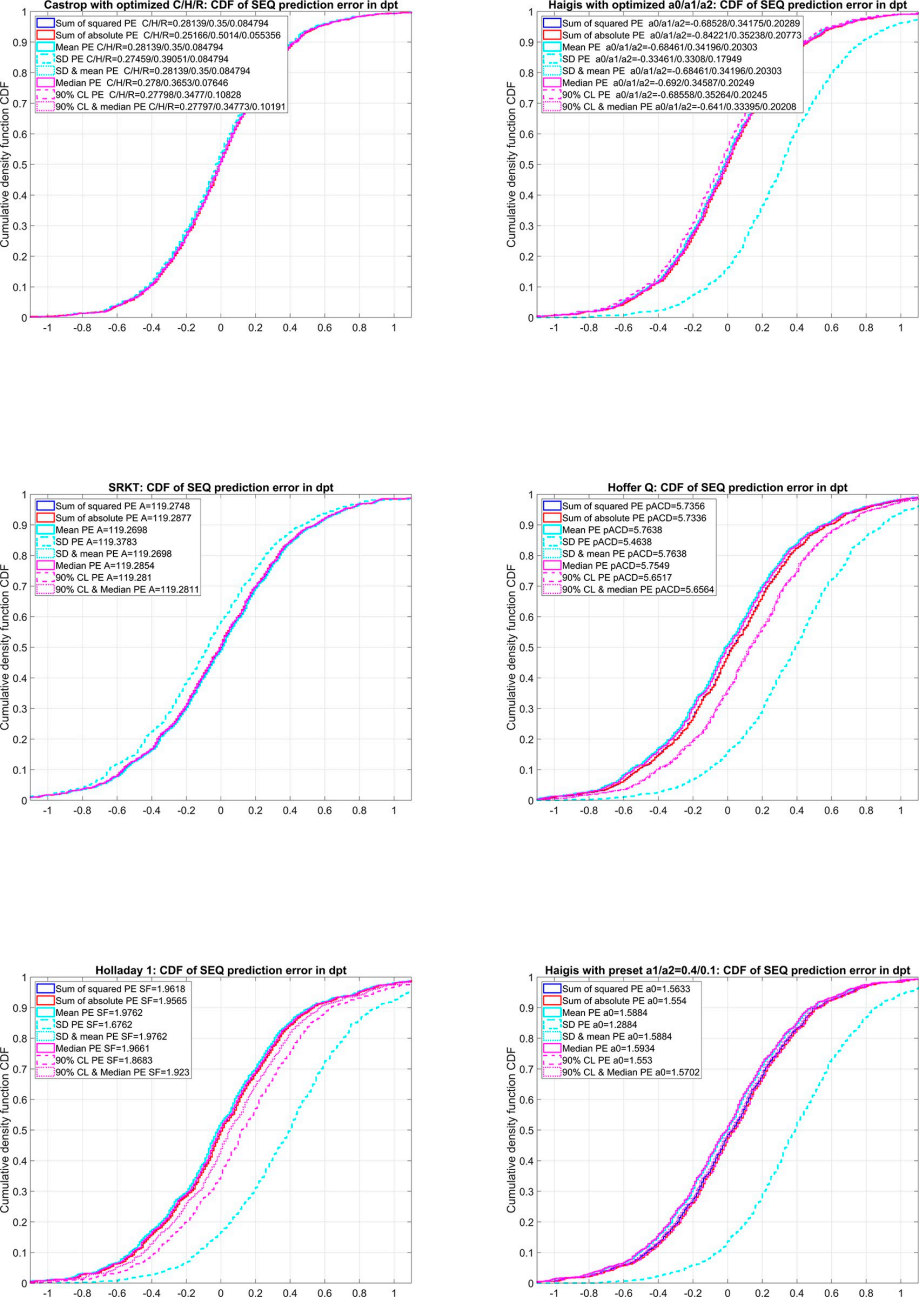
Cumulative density function (CDF) of prediction error (PE, achieved spherical equivalent–formula predicted spherical equivalent) for the SRKT, Hoffer Q, Holladay1, Haigis (with optimised a0 and preset values a1 = 0.4 / a2 = 0.1 (Haigis1); and with optimised a0 / a1 / a2 constant triplet (Haigis3)), and Castrop formula. Formula constants were optimised to minimise the sum of squared prediction errors, the sum of absolute prediction errors, the mean prediction error, standard deviation (SD) of prediction error, a combination of mean and standard deviation of prediction error (SD & mean), median prediction error, the 90% confidence interval (CL) of prediction error, and a combination of median and 90% confidence interval of prediction error (90% CL & median). The respective formula constants used for calculating the PE are shown in the graphs. Formula constant optimisation for SD or for 90% CL without considering the mean or median may in some formulae lead to a significant shift in the CDF for PE, as indicated by the dashed cyan and dashed magenta lines, whereas the optimisations for sum of squared PE, sum of absolute PE, mean PE, SD & mean PE, and 90% CL & median PE yield consistent results.

**Fig 1** shows the cumulative distribution function (CDF) of the prediction error (difference between the achieved SEQ from the formula predicted SEQ) for the 6 lens power calculation formulae (SRKT, Hoffer Q, Holladay 1, Haigis with preset a1/a2 and optimisation of a0 (Haigis1) and with optimisation of the constant triplet a0/a1/a2 (Haigis3), and Castrop formula).

For each formula under test, formula constant(s) were optimised to minimise the sum of squared prediction errors, the sum of absolute prediction errors, the mean prediction error, standard deviation (SD) of prediction error, a combination of mean and standard deviation of prediction error (SD & mean), median prediction error, the 90% confidence interval (CL) of prediction error, and a combination of median and 90% confidence interval of prediction error (90% CL & median). From the first graph (SRKT) we see that the CDFs for the prediction error are almost consistent except for the constant optimisation for the standard deviation, which ends up with an A constant which shifts the patient refraction moderately in the direction of myopia (dashed cyan line shifted to the left). From the second graph (Hoffer Q) we see that the CDFs for the prediction error are mostly consistent except for the constant optimisations for the standard deviation and for the confidence interval, both of which end up with a pACD constant with a moderate to severe shift of the patient refraction in the direction of hyperopia (dashed cyan / dashed magenta line shifted to the right). It can be seen from the third graph (Holladay 1) that, similar to the Hoffer Q formula, the CDFs for the prediction error are mostly consistent except for the constant optimisation for the standard deviation and the confidence interval, both of which end up with a surgeon factor SF which moderately or severely shifts the patient refraction in the direction of hyperopia (dashed cyan / dashed magenta line shifted to the right). The fourth graph (Haigis1) shows that the CDFs for the prediction error are mostly consistent except for the constant optimisation for the standard deviation, which ends up with an a0 which severely shifts the patient refraction in the direction of hyperopia (dashed cyan line shifted to the right). The fifth graph (Haigis3) shows that–similar to the Haigis1 formula–the CDFs for the prediction error are mostly consistent except for the constant optimisation for the standard deviation, which ends up with a a0/a1/a2 constant triplet which severely shifts the patient refraction in the direction hyperopia (dashed cyan line shifted to the right). And last but not least, from the sixth graph (Castrop) we see that the CDFs for the prediction error all are consistent. This means that all of the optimisation metrics tested in this study result in constant triplets C/H/R that do not induce systematic offsets in the formula predicted refraction.

## Discussion

Numerous formulae for calculation of intraocular lens power have been proposed in the last 20 years. In contrast to the basic formulae of Fyodorov [11] or Gernet [12] or the classical formulae of Sanders, Retzlaff and Kraff (SRKT), Hoffer (Hoffer-Q), Holladay (Holladay1) or Haigis (simplified Haigis with 1 optimised constant and Haigis formula with 3 optimised constants) [1, 4–9, 14], most of the formula authors nowadays do not disclose or publish the calculation strategy. At best they offer WEB based applications or software solutions for calculating the lenses. Such software tools do not allow batch calculations on a large set of patient data. Today, classical formulae are increasingly being replaced by ‘modern’ calculation strategies such as the Barrett Universal II, Kane, Pearl, EVO, VRF/VRF-G, Hill RBF or T2 formula in many countries of the world [2, 16]. To compare the prediction performance with other formulae it is necessary to enter the data from preoperative data (biometry), intraoperative data (lens power) and postoperative data (manual refraction after 4 weeks to 6 months) manually, introducing a large risk of transcription errors. Additionally, a systematic optimisation of constants is not possible for undisclosed formulae [15, 19, 20]. Obtaining the appropriate formula constant is mostly achieved by trial and error e.g. by varying the constants in the calculation scheme to eliminate the mean or median prediction error.

Currently there are no generally accepted guidelines or recommendations for formula constant optimisation [15, 20, 29]. Where a formula is fully disclosed, and in the simple case of a formula with a single constant, it would be possible to reorganise the formula to solve for the formula constant (for each clinical case), and from the distribution of the formula constants the clinician could select statistical metrics such as the arithmetic or geometric mean or the median. However, back-calculating the prediction error with such constants does not necessarily yield the best formula performance in terms of least root mean squared or least mean absolute error or zeroing of the mean or median of the prediction error [19]. In the case of undisclosed formulae or formulae with more than 1 constant there is no straightforward technique for back-tracing the appropriate formula constant(s) for an individual case. In the best case, when all formula constants together describe a linear superposition of a parameter for which the formula could be solved, (e.g. the effective lens position ‘d’ in the Haigis formula) a linear regression technique could be used to derive the regression coefficients (for the Haigis formula a0 as intercept, and a1 / a2 as coefficients for the phakic ACD / AL) for the dataset [19]. In some studies, when investigating the performance of a formula and comparing to other formulae, the formula constants are not optimised at all. Instead, the prediction error calculated with a given preset formula constant is zeroed for its mean or median [15, 20, 30] to analyse performance metrics such as mean absolute or root mean squared prediction error, the standard deviation, or confidence intervals of the distribution. However, such techniques do not yield proper results for the formula performance, as statistical metrics such as standard deviation or confidence intervals can change depending on the formula constant used for predicting the refractive outcome. When zeroing the mean or median prediction error, the entire distribution of the prediction error is simply shifted to centre it on a zero prediction error, which is rather different to the distribution of the prediction error when the formula constant is optimised.

A systematic optimisation of formula constants for statistical metrics of the refractive outcome requires more advanced techniques than simple reorganisation of the formula to solve for the formula constant [18]. In engineering, nonlinear iterative techniques are typically used to derive the best formula constant(s) in terms of minimising any classical statistical metrics of the prediction error. The requirements for such nonlinear techniques are: reliable and fast convergence with all datasets after a couple of iterations, robustness in a way that they result in global, rather than local, minima, and a straightforward implementation with a programming code.

In the present paper, we have used classical statistical metrics to show the capability of nonlinear iterative optimisation strategies: the sum of squared prediction error, the sum of absolute prediction error, the mean, the standard deviation of the prediction error distribution, a combination of the standard deviation of the prediction error distribution and the mean prediction error, the median prediction error, the 90% confidence interval of the prediction error, and a combination of the 90% confidence interval of the prediction error and the median prediction error. In general, such nonlinear iterative optimisation strategies are not restricted to classical metrics [28], and any continuous metrics could be used for the merit function. For statistical metrics, sum of squares and sum of absolute prediction error, special techniques which have a fast and reliable convergence to a global minimum of the merit function have been developed. Especially for minimising the sum of squared prediction error, which is equivalent to the minimisation of the root mean squared prediction error, the Levenberg-Marquardt algorithm [23, 24] is well-known in engineering disciplines to show excellent performance and robustness. For minimisation of other statistical metrics, other optimisation techniques such as the interior point methods [26, 27] or the simplex algorithm [25] are used. However, in general, if the merit function to be minimised is a ‘good-natured problem’ with a global convexity [25, 28] and without local minima, any iterative optimisation algorithm will result in a good solution for the formula constant (but with different numbers of iteration cycles and function evaluations). For this study we used the Levenberg-Marquardt algorithm [23, 24] for optimising the constant(s) for the root mean squared and the mean absolute error, and for all other metrics we used the interior point method [26, 27]. The iterative algorithms under test showed stable convergence for all formulae and for all optimisation metrics.

We see from our results that formula constant optimisation for the root mean squared and mean absolute prediction error, and also for the mean and median prediction error yields mostly consistent results for all formulae. **Fig 1** shows us that the cumulative distribution functions (CDFs) as a measure of the formula performance with this optimisation are quite coherent, and there are no systematic differences in the shape or the horizontal position of the CDFs. However, looking more in detail at the respective formula constants for the Haigis3 and the Castrop formula, both of which use constant triplets, it is obvious that the individual constants in the triplet can vary significantly (e.g. for the Castrop formula: C/H/R with optimising for SoSPE 0.2814/0.3500/0.0848 vs. 0.2517/0.5014/0.0554 with optimising for SoAPE) without significantly affecting the distributions of the prediction error [19]. All of the constant triplets work quite well when calculating for eyes with ‘normal’ biometrical parameters, but if applying these constant triplets to extreme values of biometric parameters (highly myopic/hyperopic eyes or with extremely flat or steep corneal curvatures), the resulting formula predicted refraction can vary significantly between the different constant triplets.

What can also be seen from **Fig 1** is that the standard deviation of the prediction error distribution and the 90% confidence interval of the prediction error as statistical metrics for formula constant optimisation do not yield the best results in the overall performance. In particular, optimising for the standard deviation of the prediction error without considering the mean prediction error shows a systematic offset in the CDF graphs, indicating that with the formula optimised constants having the smallest mean prediction errors, the standard deviation of the prediction error is not smallest. Only with the Castrop formula are all of the optimisation strategies more or less consistent. This is mostly due to the internal structure of the formula, where one of the formula constants (R) is used as a systematic offset value for the formula predicted refraction, mainly to account for different lane distances in the refraction measurements. With this constant the CDF may be centred using the constant optimisation strategy without changing the ‘shape’ of the CDF. However, optimising formula constants for the 90% confidence interval does not yield acceptable results for all of the formulae under test. In particular, the CDF for the Hoffer Q and the Holladay 1 formula is not centred and indicates some systematic offset. This means that optimising for the confidence interval without considering the median prediction error may provide constant(s) with a narrow distribution, but probably with a systematic offset in the prediction error. To overcome this problem we completed our optimisation strategies using a compromise of using combinations of the standard deviation of the prediction error distribution and the mean prediction error to ensure that the CDF is centred and the standard deviation is considered in the optimisation (optimising for SDMPE). Accordingly, we completed our optimisation strategies using a compromise of using combinations of the 90% confidence interval of the prediction error and the median prediction error to ensure that the CDF is centred and the confidence interval is considered in the optimisation (optimising for CLMEDPE). Both optimisations show a good performance in terms of low systematic offset of the prediction error and a narrow CDF.

The results of the classical performance indicators for lens power calculation concepts are summarised in **Table 3**. The mean error mostly accounts for a systematic shift towards either myopia or hyperopia in the formula predicted refraction, and the standard deviation accounts for the width of the distribution if a (parametric) normal distribution is considered. The median and the lower and upper boundaries of the 90% confidence interval (together with the width of the 90% confidence interval) refer to the (nonparametric) metrics of an arbitrary distribution for the prediction error. Finally, the mean absolute and the root mean squared prediction error are classical measures in the literature to evaluate the performance of formula predicted refractions, but both are significantly affected by systematic offsets in the prediction error. This means that they are not proper measures of the consistency of the results. The listing implies that the optimisation strategies worked quite well: where we optimised for the SoSPE the resulting root mean squared error (RMS) is the lowest in the row for all formulae. When optimising for the SoAPE it is clear that on our dataset the mean absolute error (ABS) is the lowest in the row. Where we have optimised for the mean prediction error (MPE) or the median prediction error (MEDPE), it is can be seen that the resulting mean prediction error (MEAN) or the median prediction error (MEDIAN) is lowest in the row. When optimising for the standard deviation of the prediction error distribution (SDPE) we see that the standard deviation (SD) is lowest in the row, but at the cost of a systematic shift in mean or median formula predicted refraction (MEAN or MEDIAN). Accordingly, when optimising for the 90% confidence interval, the width of the confidence interval (90% CL) is lowest in the row at the cost of the mean or median value (MEAN and MEDIAN) which are systematically shifted, at least in the Hoffer Q and the Holladay formula.

**In conclusion**, this study describes techniques for optimising formula constants on a clinical dataset using modern nonlinear iterative optimisation strategies. These optimisation strategies are generally capable of dealing with all disclosed lens power calculation formulae having one or more formula constants, and with any statistical metric of any outcome measure. The applicability of this constant optimisation technique is shown for the SRKT, Hoffer Q, Holladay 1, Haigis, and Castrop formula on a clinical dataset with 888 data from 1 clinical centre treated with the Hoya Vivinex intraocular lens. The prediction error, defined as the difference between the postoperatively achieved and the formula predicted spherical equivalent power of refraction, was used as a merit function. Optimisations for the root mean squared and mean absolute prediction error and for the mean and median prediction error yielded mostly consistent performance results, but optimisations for the standard deviation of the prediction error distribution or the confidence interval may result in a narrow distribution but with some systematic offset in the formula predicted refraction which is not appropriate in a clinical setting.
